# Variation in activity levels amongst dogs of different breeds: results of a large online survey of dog owners from the UK

**DOI:** 10.1017/jns.2017.7

**Published:** 2017-04-17

**Authors:** Emily Pickup, Alexander J. German, Emily Blackwell, Mark Evans, Carri Westgarth

**Affiliations:** 1 Institute of Veterinary Science, University of Liverpool, Neston, UK; 2 Institute of Ageing and Chronic Disease, University of Liverpool, Neston, UK; 3 School of Clinical Veterinary Science, University of Bristol, Langford, UK; 4 Independent Veterinary Consultant, Guildford, UK; 5 Institute of Infection and Global Health, University of Liverpool, Liverpool, UK

**Keywords:** Obese dogs, Canine nutrition, Physical activity, Dog walking, KC, Kennel Club

## Abstract

Regular physical activity is an important means of promoting health, both in people and their pets. Walking is the most common method used for dogs, but there is a lack of clarity on how much daily activity different breeds of dog require. Data from an online survey of UK dog owners were collected between June and August in 2014. The University of Liverpool Ethics Committee approved the project, and owners consented to data use. The initial dataset (17 028 dogs) was first cleaned to remove erroneous data, and then edited to remove mixed breed dogs, leaving a total of 12 314 dogs from known pedigree breeds. Other information collected included sex, age, neuter status, breed, and amount and frequency of exercise. Exercise frequency and duration were estimated across different breeds, and compared with Kennel Club recommendations, using *χ*^2^ tests and binary logistic regression. The online survey data indicated differences amongst breeds in the amount of walking reported (*P* < 0·001). Afghan hounds were the least exercised breed, whilst breeds reportedly exercised most included: English setter, foxhound, Irish setter and Old English sheepdog. Gundogs were most likely to be walked once per d or more (*P* < 0·001), whilst smaller dogs were more likely to meet their UK Kennel Club guidelines for dog walking (*P* < 0·001). The frequency of dog walking varies both within and amongst breeds, and many do not currently receive the recommended amount of exercise. This may constitute a canine welfare problem and also have an impact on the physical activity levels of their owners.

Regular physical activity is an important means of promoting health in people^(^^1^^)^. A similar recommendation is made in dogs, but there is a lack of clarity on how much different breeds of dog require, and no current evidence-based guidelines are available^(^^2^^)^. The US Department of Agriculture recommends that dogs should have at least 30 min of exercise per d; however, this recommendation mainly came from anecdotal evidence and expert opinion guidelines^(^^3^^)^. In the UK, the Kennel Club (KC) has published recommendations regarding activity requirements for different breeds^(^^4^^)^. However, once again, these recommendations are not taken from scientific evidence but based on opinions from the breed clubs. There is generally a positive association between size of the dog and exercise recommendations, i.e. the bigger the dog, the more exercise it is perceived to require.

Before recommendations can be made regarding the optimal amount of activity different breeds require, it is first necessary to determine current activity levels and what determines them. In a recent review of the correlates of dog walking, there was some evidence of smaller dogs being walked less than bigger dogs, although the association was not clear^(^^5^^)^. There was also no evidence available regarding the influence of specific breed beyond broad type groupings; this was probably due to the size of the dataset available, which limited the ability to perform individual breed analyses. The aims of this study were to investigate differences in the amount and frequency of exercise amongst dogs of different breed, and to determine the proportion of dogs within each breed that meet current recommendations.

## Methods and materials

### Study design

An online survey of UK dog owners was conducted between June and August in 2014, in association with the broadcast of a three-part Channel 4 television documentary series, ‘Dogs: Their Secret Lives’. Information was gathered from owners about the signalment of their dog (age, sex, neuter status and breed), body weight, whether or not the dog was overweight, lifestyle, activity and behaviour. The main questions considered in the current study are those regarding activity, breed and other signalment details. The data on exercise have also been used in a separate study examining the associations between exercise and overweight status^(^^6^^)^, whilst the questions relating to behaviour are reported elsewhere^(^^7^^)^.

For owner responses, most questions involved either checking boxes or using drop-down menus. The main questions used in the current study were on breed and activity. Breed of dog was indicated using a drop-down list of UK breeds. For exercise frequency, the question asked was ‘How often do you exercise your dog outside of your home or garden?’ and respondents could select: ‘more than once a day’, ‘once a day’, ‘4–6 times per week’, ‘1–3 times per week’ or ‘never’. For exercise duration, the question asked was ‘Each time you exercise your dog how long is it for?’ and respondents could select: ‘over an hour’, ‘30 minutes to an hour’, ‘11–30 minutes’ and ‘0–10 minutes’. Respondents were also asked if the dog is let off the lead (yes/no).

The University of Liverpool Ethics Committee approved the study, and all owner participation was voluntary, whereby owners who wished to complete the survey logged onto the Channel 4 website. Further, owners gave permission for their data to be used, in a fully anonymised form (i.e. any client-identifying data removed), and for the results to be publicised both on the television shows and online. They were not required to answer questions that they did not wish to answer. To be eligible for inclusion in the data analysis part of the study, dogs had to be from a pedigree breed (based upon the breeds officially recognised by the UK KC)^(^^4^^)^ and questionnaire information needed to be complete, i.e. all questions used in the current study needed to be answered.

### Data handling and statistical analysis

All data were first entered into a computer spreadsheet (Excel version 14; Microsoft), to enable data manipulation prior to statistical analysis. Given that the study involved a direct comparison of exercise amongst pedigree breeds, the data from all mixed breed dogs were removed. Breed data were then further categorised into breed-specific groups (e.g. pastoral, gundogs, hounds, terriers, toy, utility or working) and by size (e.g. small, medium or large), according to The UK KC classification^(^^4^^)^. Unlike for the related papers^(^^6^^,^^7^^)^, data from dogs under 2 years of age were eligible for inclusion in the analysis for this study. Outcome variables of interest included: frequency of exercise outside of the home/garden (see categories above); walked once per d or more (yes/no); duration of usual exercise (see categories above); and met UK KC guidelines of exercise for that breed (yes/no). The latter outcome was created by comparing each dog's reported exercise with the activity requirements recommended by the UK KC for different breeds^(^^4^^)^. Data analysis was conducted in Minitab 17 and IBM SPSS Statistics 22. We used *χ*^2^ tests to compare proportions of dogs amongst exercise categories. Given that multiple comparisons were performed, a modified Bonferroni correction was applied^(^^8^^)^. This correction effectively meant that statistical significance was only considered when *P* < 0·0042. Caution should be taken when interpreting findings as even in such a large dataset, numbers in each breed were often small, hence deeper statistical analysis was not conducted.

## Results

### Final dataset

Data were available from questionnaires of 17 028 dogs, of which 9480 were male (56 %) and 7548 (44 %) were female. Of the dogs, 2849 (17 %) were <1 year of age, 4470 (26 %) were 1–3 years old, 8206 (48 %) were 3–10 years old, and 1486 (9 %) were >10 years old. A total of 12 314 were pedigree dogs, comprising seventy-one separate breeds, and the remaining 4714 were mixed-breed dogs (which were not included in the data analysis).

### Exercise frequency

There was a difference amongst dogs of different breeds for the likelihood of being exercised at least once per d (*P* < 0·001; Table 1). The breeds which were most likely to be exercised once per d or more were English setter (20/20, 100 %), foxhound (20/20, 100 %), Irish setter (24/24, 100 %), Old English sheepdog (19/19, 100 %) and Hungarian Vizla (87/89, 98 %). The breeds least likely to be exercised once per d or more were Afghan hound (5/10, 50 %), papillon (11/19, 59 %), Pyrenean mountain (3/5, 60 %), bloodhound (3/5, 60 %) and Chihuahua (101/162, 62 %). The frequency of exercise also varied amongst dogs of different breed groups (*P* < 0·001; Table 2). Dogs in the gundog (3869/4325, 90 %), pastoral (1380/1585, 88 %) and hound (789/924, 85 %) groups were most likely to be exercised once per d or more; in contrast, dogs of the terrier (2257/2793, 81 %) and toy groups (603/814, 74 %) were least likely to be exercised once per d or more. There was a difference between the size of the dog and whether it received exercise once per d or more (*P* < 0·001), with large dogs (4380/5006, 88 %) being more likely to receive exercise once per d or more compared with medium (4105/4832, 85 %) and small dogs (1948/2476, 79 %). There were also significant differences in the frequency of exercise amongst dogs of different ages (older dogs < younger dogs; *P* < 0·001) and neuter status (entire dogs < neutered dogs, *P* < 0·001)

**Table 1.** Exercise frequency, exercise length, daily exercise, off-the-lead exercise and whether Kennel Club (KC) guidelines were met in each breed of dog (Numbers and percentages)

			Exercise frequency										Exercise length											
	Total		Never		1–3/week		4–6/week		1/d		>1/d		0–10 min		11–30 min		30–60 min		>1 h		Off lead in public		Met KC guideline*	
Breed	*n*	%	*n*	%	*n*	%	*n*	%	*n*	%	*n*	%	*n*	%	*n*	%	*n*	%	*n*	%	*n*	%	*n*	%
Afghan hound	10	0·1	2	20	1	10	2	20	1	10	4	40	2	20	2	20	3	30	3	30	6	60	2	20
Airedale terrier	23	0·1	0	0	2	9	2	9	6	26	13	56	0	0	6	26	10	44	7	30	21	91	18	78
Akita	55	0·3	1	2	6	11	3	6	12	22	33	60	0	0	6	11	39	71	10	18	23	42	4	7
Alaskan Malamute	52	0·3	0	0	5	10	4	8	15	29	28	54	0	0	10	19	22	42	20	38	21	40	11	21
Basset hound	70	0·4	1	1	12	17	3	4	23	33	31	44	5	7	27	39	22	31	16	23	47	67	44	63
Beagle	225	1·3	1	0	11	5	19	8	77	34	117	52	2	1	42	19	133	59	48	21	152	68	21	9
Bearded collie	58	0·3	0	0	2	3	0	0	19	33	37	64	1	2	12	21	30	52	15	23	48	83	54	93
Bedlington terrier	23	0·1	0	0	3	13	1	4	5	22	14	61	1	4	6	26	10	44	6	26	18	78	18	78
Bernese mountain dog	25	0·1	0	0	0	0	2	8	9	36	14	56	0	0	10	40	13	52	2	8	18	72	20	80
Bichon frise	147	0·9	2	1	17	12	13	9	50	34	65	44	10	7	77	52	47	32	13	9	75	51	115	78
Bloodhound	5	0·0	0	0	0	0	2	40	1	20	2	40	1	20	2	40	1	20	1	20	3	60	0	0
Border collie	840	4·9	12	1	39	5	42	5	228	27	519	62	10	1	159	19	471	56	200	24	699	83	118	14
Border terrier	318	1·9	5	2	19	6	17	5	105	33.	172	54	5	2	79	25	171	54	63	20	228	72	255	80
Boston terrier	23	0·1	1	4	1	4	0	0	8	35	13	57	1	4	10	44	10	44	2	9	19	83	17	74
Boxer	218	1·3	0	0	9	4	17	8	71	37	121	56	2	1	57	26	119	55	40	18	162	74	18	8
Bull terrier	80	0·5	0	0	9	11	10	13	30	38	31	39	0	0	26	32	39	49	15	19	44	55	52	65
Bulldog	91	0·5	4	4	9	10	8	9	43	47	27	28	6	7	37	41	40	44	8	9	64	70	49	54
Bullmastiff	56	0·3	1	2	8	14	7	12	15	27	25	45	0	0	18	32	32	57	6	11	32	57	2	4
Cairn terrier	87	0·5	0	0	8	9	6	7	17	20	56	64	1	1	33	38	47	54	6	7	59	68	67	77
Cavalier King Charles spaniel	334	2·0	6	2	36	11	31	9	107	32	154	46	14	4.	137	41	155	46	28	8	249	75	213	64
Chihuahua	162	1·0	6	4	36	22	19	12	57	35	44	27	12	7	74	46	60	37	16	10	86	53	101	62
Chinese crested	17	0·1	0	0	0	0	2	12	6	35	9	53	1	6	5	29	10	59	1	60	12	71	15	82
Chow chow	11	0·1	0	0	1	9	1	9	3	27	6	54	0	0	7	64	4	36	0	0	3	27	7	64
Cocker spaniel	833	4·9	2	0	42	5	42	5	244	29	503	60	6	1	193	23	494	59	140	17	683	82	704	84
Collie (rough/smooth)	82	0·5	2	2	4	5	4	5	24	29	48	58	1	1	19	23	46	56	16	20	68	83	67	82
Dachshund	137	0·8	3	2	20	15	14	10	56	41	44	32	9	7	57	42	63	46	8	6	90	66	100	73
Dalmatian	99	0·6	0	0	6	6	5	5	38	38	50	50	1	1	10	10	53	54	35	35	77	78	13	13
Dobermann	79	0·5	0	0	9	11	2	2	30	38	38	48	0	0	12	15	46	58	21	27	57	72	8	10
Dogue de Bordeaux	28	0·2	0	0	3	11	1	4	10	36	14	50	0	0	10	36	15	54	3	11	18	64	20	71
English setter	20	0·1	0	0	0	0	0	0	8	40	12	60	0	0	3	15	9	45	8	40	16	80	2	10
English springer spaniel	702	4·1	5	1	43	6	38	5	168	24	448	64	11	2	136	19	414	59	141	20	605	86	75	11
Flat coated retriever	68	0·4	0	0	0	0	3	4	28	41	37	54	1	2	9	13	43	6	15	22	59	87	8	12
Fox terrier	46	0·3	1	2	3	6	0	0	19	41	23	50	1	2	19	41	17	37	9	20	33	72	34	74
Foxhound	3	0·0	0	0	0	0	0	0	1	33	2	67	0	0	0	0	2	67	1	33	2	67	0	0
French bulldog	79	0·5	0	0	5	6	10	13	34	43	30	38	0	0	37	47	38	48	4	5	59	75	52	66
German pointer	107	0·6	0	0	7	7	1	1	23	22	76	71	0	0	6	5	59	55	42	39	94	88	28	26
German shepherd dog	511	3·0	5	1	54	11	30	6	155	30	267	52	7	1	115	22	273	53	116	23	364	71	56	11
Giant Schnauzer	7	0·0	0	0	1	14	0	0	2	29	4	57	0	0	0	0	5	71	2	27	6	86	0	0
Golden retriever	400	2·3	1	0	21	5	18	4	128	32	232	58	3	1	80	20	238	60	79	19·8	342	86	46	12
Great Dane	35	0·2	1	3	4	11	7	20	10	29	13	37	3	9	10	29	19	54	3	9	27	77	1	3
Greyhound	228	1·3	1	0	11	5	5	2	54	24	157	69	4	2	106	46	98	43	20	9	129	57	187	82
Hungarian Vizsla	89	0·5	0	0	1	1	1	1	17	19	70	79	0	0	5	6	58	65	26	29	78	88	15	17
Irish setter	52	0·3	0	0	0	0	0	0	20	39	32	62	0	0	8	15	33	64	11	21	38	73	4	8
Irish terrier	24	0·1	0	0	0	0	1	4	8	33	15	62	0	0	7	29	12	50	5	21	20	83	21	88
Jack Russell terrier	929	5·5	17	2	91	10	72	8	276	30	473	51	30	3	324	35	465	50	110	12	628	68	656	71
Labrador retriever	1975	11·6	13	1	93	5	114	6	552	28	1203	61	29	2	470	24	1158	57	318	16	1722	87	154	8
Lhasa apso	127	0·7	0	0	15	12	10	8	37	29	65	51	7	6	58	46	57	45	5	4	71	56	102	80
Mastiff	38	0·2	0	0	2	5	5	13	14	37	17	45	1	3	13	34	18	47	6	16	28	74	28	74
Miniature Schnauzer	199	1·2	1	0	7	4	9	4	52	26	130	65	2	1	77	39	99	50	21	11	151	76	163	82
Newfoundland	37	0·2	2	5	1	3	3	8	15	40	16	43	2	5	14	38	21	57	0	0	22	60	24	65
Old English sheepdog	19	0·1	0	0	0	0	0	0	5	26	14	74	0	0	2	10	11	58	6	32	18	95	5	26
Papillon	19	0·1	0	0	7	37	1	5	4	21	7	37	0	0	9	47	9	48	1	5	12	63	11	58
Pekingese	3	0·0	0	0	1	33	0	0	2	67	0	0	(0	0	2	67	1	33	0	0	2	67	2	67
Pomeranian	33	0·2	1	3	7	21	1	3	15	46	9	27	5	15	12	36	14	42	2	6	16	48	24	73
Poodle	110	0·6	0	0	10	9	6	6	36	33	58	53	7	6	35	32	54	49	14	13	82	74	78	71
Pug	99	0·6	1	1	14	14	10	10	28	28	46	46	2	2	49 50		39	39	9	9	68	69	74	75
Pyrenean mountain dog	5	0·0	1	20	0	0	1	20	3	60	0	0	0	0	1	20	1	20	3	60	2	40	3	60
Rhodesian ridgeback	67	0·4	1	2	2	3	4	6	20	30	40	60	2	3	11	16	38	57	16	23·9	54	81	10	15
Rottweiler	169	1·0	2	1	17	10	15	9	57	34	78	46	2	1	49	29	86	51	32	19	117	69	12	7
Saluki	33	0·2	0	0	0	0	2	6	13	39	18	54	0	0	12	36	15	46	6	18	19	58	3	9
Samoyed	46	0·3	0	0	3	6	3	6	17	37	23	50	0	0	13	28.	23	50	10	22	32	70	3	6
Shar Pei	44	0·3	0	0	8	18	1	2	15	34	20	46	2	4	17	39	18	41	7	16	21	48	30	68
Shetland sheepdog	24	0·1	0	0	0	0	3	12	9	38	12	50	0	0	4	17	15	62	5	21	19	79	21	88
Shih Tzu	195	1·1	4	2	31	16	23	12	44	23	93	48	10	5	91	47	78	40	16	8	125	64	115	59
Siberian husky	110	0·6	1	1	11	0	9	8	39	36	50	46	0	0	19	17	59	54	32	29	43	39	13	12
St Bernard	9	0·1	0	0	2	22	0	0	3	33	4	44	0	0	1	11	7	78	1	11	4	44	7	78
Staffordshire bull terrier	743	4·4	9	1	88	12	62	8	236	32	348	47	17	2	238	32	387	52	101	14	502	68	513	69
Weimaraner	79	0·5	4	5	5	6	2	2	24	30	44	56	0	0	10	13	46	58	23	29	70	89	11	14
West Highland white terrier	318	1·9	2	1	24	8	27	8	106	33	159	50	5	2	116	36	166	52	31	10	223	70	237	74
Whippet	146	1	1	1	6	4	14	10	49	34	76	52	3	2	31	21	86	59	26	18	106	73	113	77
Yorkshire terrier	179	1	3	2	29	16	23	13	55	61	69	38	8	4	92	51	74	43	5	3	103	58	99	55

* Dogs meeting KC guidelines.

**Table 2.** Exercise frequency based on age, sex, neuter status, UK Kennel Club (KC) grouping and size (Numbers and percentages)

	Exercise <1/d		Exercise ≥1/d		
Variable	*n*	%	*n*	%	P*
Age (years)					<0·0001
1–3	647	12	4594	88	
3–10	1504	16	8086	84	
>10	395	18	1784	82	
Sex					0·565
Male	1408	15	8072	85	
Female	1145	15	6403	85	
Neuter status					<0·0001
Entire	733	18	3288	82	
Neutered	1820	14	11187	86	
KC groupings^†^					<0·0001
Gundog	456	10	3869	90	
Pastoral	205	13	1380	87	
Hound	138	15	786	85	
Working	151	18	712	82	
Utility	184	18	826	82	
Terrier	536	19	2257	81	
Toy	211	26	603	74	
Size of dog^‡^					<0·0001
Small	528	21	1948	79	
Medium	727	15	4105	85	
Large	626	12	4380	88	

* Comparisons made with the *χ*^2^ test.

† KC grouping: breed group based upon UK KC classification.

‡ Size based upon UK KC classification.

### Exercise length

There was a difference (*P* < 0·001) amongst the breeds in the duration of exercise: Alaskan Malamute (42/52, 81 %), beagle (181/225, 80 %), Border collie (671/840, 80 %), cocker spaniel (687/833, 82 %), Dalmatian (88/99, 89 %), German pointer (101/107, 94 %), Hungarian Vizla (74/89, 83 %), Old English sheepdog (17/19, 89 %), Shetland sheepdog (20/24, 83 %) and Weimaraner (69/79, 87 %) were most likely to be exercised for over 1 h; in contrast, bichon frise (50/147, 34 %), greyhound (118/228, 52 %), Lhasa apso (62/127, 49 %), Pomeranian (16/33, 48 %) and Yorkshire terrier (79/179, 44 %) were some of the breeds least likely to be exercised for more than 1 h. There was also a difference (*P* < 0·001) amongst breed groups for the duration of exercise received; dogs in the gundog (3355/4325, 78 %), pastoral (1241/1585, 78 %) and working (630/866, 73 %) groups were more likely to receive exercise for half an hour or more than dogs of toy (405/814, 50 %), utility (600/810, 59 %), terrier (1768/2793, 63 %) and hound (606/924, 66 %) groups.

A positive association (*P* < 0·001) was also found between size of the dog and the duration of exercise they received. Small dogs (1369, 55 %) were less likely to receive exercise for longer than 30 min compared with medium dogs (3484, 72 %) and large dogs (3752, 75 %). Almost half (45 %) of the dogs exercised for less than 30 min were small dogs.

### Off-lead exercise

Some breeds were more commonly allowed off the lead in public (*P* < 0·001; Table 1), including: chow chow (8, 73 % let off the lead); Siberian husky (67, 61 %); Pyrenean mountain dog (3, 60 %); Alaskan Malamute (31, 60 %); and St Bernard (5, 56 %). Differences were also noted in frequency with which dogs in different breed groups were let off the lead in public (*P* < 0·001): working (555, 64 %) and toy (520, 64 %) were least likely to be let off the lead; pastoral (1250, 79 %) and gundogs (3707, 86 %) were most likely to be let off the lead; and hound (608; 66 %), utility (676, 67 %) and terrier (1898; 68 %) dogs were intermediate.

### Exercising in relation to UK Kennel Club guidelines

There was a significant difference (*P* < 0·001) between size of the dog and their KC exercise recommendations. Small dogs were recommended up to 1 h per d (1732, 70·0 %) or up to 30 min per d (744, 30·0 %); medium dogs were recommended more than 2 h per d (1767, 36·6 %) or up to 1 h per d (3065, 63·4 %); large dogs were recommended more than 2 h per d (4364, 87·2 %) or up to 1 h per d (642, 12·8 %). Thus there appears to be a positive association between size of the dog and exercise recommendations, i.e. the bigger the dog, the more exercise it is perceived to require. Based upon the information recorded, 9025 dogs in the survey (53 %) did not meet the UK KC guidelines for exercise for the respective breed, and differences amongst breeds were seen (Table 1; *P* < 0·0001). The breeds most likely to meet the KC guidelines included: bearded collie (54/58, 93 %), Chinese crested dog (15/17, 88 %); Irish terrier (21/24, 88 %); Shetland sheepdog (21/24, 88 %); and cocker spaniel (704/833, 84 %). The breeds least likely to meet the KC guidelines included: bloodhound (0/5, 0 %); giant Schnauzer (0/7, 0 %); foxhound (0/3, 0 %); great Dane (1/35, 3 %); and bullmastiff (2/56, 4 %). Even in popular breeds like the Labrador retriever only 8 % were reported to meet the guidelines for activity.

When classed into KC breed groupings, terriers were most likely to meet KC exercise recommendations (1987/2793, 71 %), whilst working (164/863, 19 %) and pastoral dogs were least likely (327/1585, 21 %) (*P* < 0·001) to meet exercise requirements. Finally, there was a difference in the proportion of dogs of different sizes that met UK KC requirements (*P* < 0·0001): small dogs (1762/2476, 71 %) were more likely to receive their daily exercise requirement than either medium (2493/4832, 52 %) or large dogs (919/5006, 18 %).

## Discussion

This is the first study to investigate differences in reported exercise between dogs of different breeds. We found differences between (and within breeds) relating to factors including: exercise frequency, exercise length, and whether or not the dog met the UK KC guidelines^(^^4^^)^. Whilst these findings indicate current exercise patterns, further work is now required to determine their significance, for instance, whether different exercise patterns can predispose to or protect from diseases such as obesity. It is suggested that an inverse correlation exists between physical activity of dogs and canine obesity^(^^9^^)^. By encouraging dog owners to meet their own recommended daily exercise, it is hoped that the health and wellbeing of pet dogs will be improved^(^^5^^)^. Further studies should also be considered not just to examine frequency and duration of exercise but also the nature of the activity undertaken. In one recent study, the majority of time during a dog walk was spent sniffing, suggesting minimal activity overall^(^^10^^)^. Therefore, future studies should also measure intensity of exercise and perhaps distance travelled, using objective methods such as accelerometry^(^^11^^)^ and global positioning system receivers^(^^12^^)^.

Differences were seen amongst different breeds in terms of the frequency of exercise. Most notably, breeds in the gundog, pastoral and hound groups were more frequently exercised than other dogs, most notably the toy and terrier groups. Similarly, duration of exercise varied amongst breed groups and, once again, those in the gundog, pastoral and hound groups were exercised for longest, whilst those in the terrier and toy breed groups were exercised least frequently. Similarly, when categorised by size, smaller dogs were exercised for a shorter time overall and less frequently than medium or larger breed dogs, although some notable exceptions occurred (e.g. both Afghan and foxhounds were amongst the least and most frequently exercised) that require further investigation. These findings support those of a recent review of the correlates of dog walking, which also found evidence for smaller dogs being walked less than bigger dogs^(^^5^^)^. Such differences in exercise pattern might reflect differences in capabilities for exercising amongst breeds, but might also be related to owner factors and geographical location. For example, many owners choose breeds based upon their lifestyle, and owners might select smaller breeds if they are concerned they will not have enough time to exercise a more athletic breed. Geographical location might dictate choice of breed, with more athletic breeds being more commonly chosen when living in rural locations. Unfortunately, given that data were fully anonymised, it was not possible to assess owner or geographical effects, and further studies, likely including in-depth qualitative investigation, are required.

Although no evidence-based guidelines exist regarding the exercise that dogs in each breed require each day, general recommendations are provided by many canine organisations, such as the UK KC^(^^4^^)^. For example, the UK KC provides guidance by breed regarding the recommended daily exercise, with recommendations varying amongst breeds between >30 min/d to >2 h/d. In this respect, the recommended exercise for many of the smaller breed dogs (e.g. Chihuahua, Lhasa apso, papillon, Pomeranian and Yorkshire terrier) is >30 min/d, although >1 h/d is recommended for some small breed dogs (e.g. Border terrier, Lhasa apso and pug). The typical recommendation for many of the medium breeds (e.g. cavalier King Charles spaniel, cocker spaniel, miniature Schnauzer, Staffordshire bull terrier) is >1 h/d, whilst the typical recommendation for larger breeds (e.g. bullmastiff, Dobermann, English setter, great Dane, Labrador retriever, Rottweiler, Saluki and Samoyed) is >2 h/d, although >1 h/d is recommended for some larger breeds (e.g. greyhound), whilst >2 h/d is recommended for some of the more active medium-size breeds (e.g. Border collie and English springer spaniel). Perhaps the most concerning observation of the present study was the fact that only half of the dogs studied received the recommended amount of exercise. In contrast with the frequency and duration of exercise reported, it was more often dogs in many of the larger breeds that did not meet the recommendations. This suggests that it is particularly challenging for owners to ensure that their dog receives >2 h/d of exercise. In light of this, evidence-based recommendations are urgently needed to determine whether the 2-h recommendation is necessary; if it is, better education of prospective dog owners is required to ensure that they understand activity requirements of different breeds, and the particular challenges faced with the larger breeds.

A number of limitations, in addition to those discussed above, should be considered when interpreting the results. First, whilst the use of a voluntary online survey increased the number of responses, it was a convenience sample and, therefore, there are concerns regarding selection bias. In this respect, we cannot be certain that the participants were truly representative of the UK dog population as a whole; it might be that owners who exercise their dog more were more willing to participate than those who did not. The use of self-reports of exercise is subjective, and can be unreliable^(^^10^^)^. Third, in responding, owners could only select from a small number of categories, and owners might have had trouble choosing between categories, not least if they exercised their dogs irregularly in terms of frequency and duration. The use of categories also meant that it was not possible to investigate observations more fully. Therefore, these observations should be considered to be preliminary and further studies are recommended to explore the issue of activity amongst breeds in more detail.

### Conclusions

Exercise patterns of dogs in the UK vary. The finding that half of dogs in the study were not receiving the recommended activity level is concerning. Further work is required to achieve a better understanding of what physical exercise is actually required within breed, and how this relates to health and owner perception.
