# Variation in activity levels amongst dogs of different breeds: results of a large online survey of dog owners from the UK – CORRIGENDUM

**DOI:** 10.1017/jns.2021.29

**Published:** 2021-10-21

**Authors:** Emily Pickup, Alexander J. German, Emily Blackwell, Mark Evans, Carri Westgarth

**Affiliations:** 1 Institute of Veterinary Science, University of Liverpool, Neston, UK; 2 Institute of Ageing and Chronic Disease, University of Liverpool, Neston, UK; 3 School of Clinical Veterinary Science, University of Bristol, Langford, UK; 4 Independent Veterinary Consultant, Guildford, UK; 5 Institute of Infection and Global Health, University of Liverpool, Liverpool, UK

This article was published with errors in the first sentence of the section ‘Off-lead exercise’ on page 5. The correct sentence is below.

*Off-lead exercise*

Some breeds were **less** commonly allowed off the lead in public (P < 0⋅001; Table 1), including: chow chow (8, 73% **not** let off the lead); Siberian husky (67, 61%); Pyrenean mountain dog (3, 60%); Alaskan Malamute (31, 60%); and St Bernard (5, 56%).

The authors apologise for the error.
